# Integrated bioinformatic pipeline using whole-exome and RNAseq data to identify germline variants correlated with cancer

**DOI:** 10.1016/j.xpro.2022.101273

**Published:** 2022-04-04

**Authors:** Divya Sahu, Ajay Chatrath, Aakrosh Ratan, Anindya Dutta

**Affiliations:** 1 Department of Genetics, University of Alabama at Birmingham, Birmingham, AL 35294, USA; 2 Department of Biochemistry and Molecular Genetics, University of Virginia, Charlottesville, VA 22903, USA; 3 Center for Public Health Genomics, University of Virginia, Charlottesville, VA 22908, USA

**Keywords:** Bioinformatics, Cancer, Genetics, Genomics, Sequencing, RNAseq, Systems biology

## Abstract

Germline Variants (GVs) are effective in predicting cancer risk and may be relevant in predicting patient outcomes. Here we provide a bioinformatic pipeline to identify GVs from the TCGA lower grade glioma cohort in Genomics Data Commons. We integrate paired whole exome sequences from normal and tumor samples and RNA sequences from tumor samples to determine a patient’s GV status. We then identify the subset of GVs that are predictive of patient outcomes by Cox regression.

For complete details on the use and execution of this protocol, please refer to Chatrath et al. (2019) and Chatrath et al. (2020).

## Highlights

• Integration of whole-exome and RNA sequences to determine Germline Variants (GVs)
• Whole-exome and RNA sequences from tumors resolve low coverage issue in normal samples
• High correlation of GV allele frequencies between patient data and the GnomAD database
• GVs predict patient cancer outcome

## Graphical abstract

**Figure:**
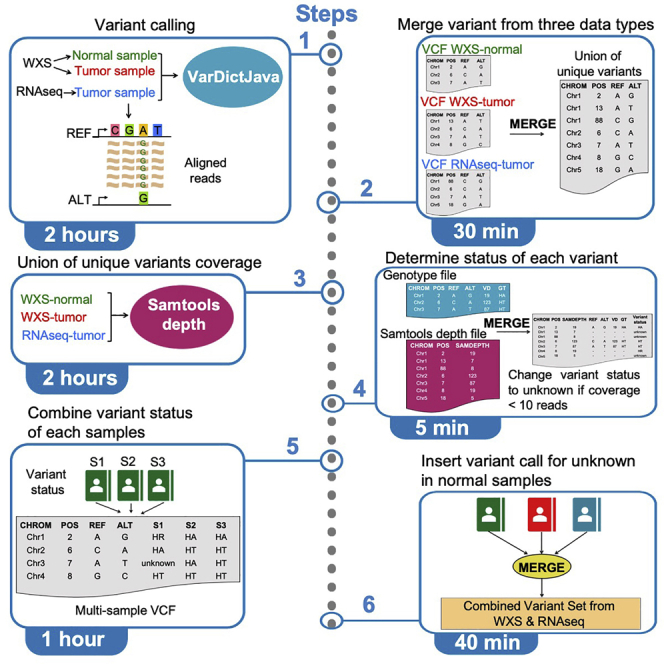

## Before you begin

### Clone the protocol directory

**Timing: 5 min**

The scripts and test data sets required to run this protocol are available on the GitHub repository

https://github.com/ds21uab/STAR_protocols_GV_calling.git.

1. Open the Linux command-line interface. Copy and paste the command below to clone protocol directory from the GitHub repository:

>git clone

https://github.com/ds21uab/STAR_protocols_GV_calling.git

### Set path for the protocol directory

**Timing: 1 min**

2. Save path for the ‘STAR_protocols_GV_calling’ directory in a local or online Linux cluster.
  a. To determine the exact location of the ‘STAR_protocols_GV_calling’ directory using command-line, type command pwd from the directory where “STAR_protocols_GV_calling” is located
    >pwd
    The output should look something like this

**Figure:**
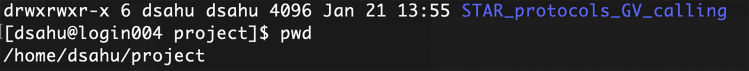
    The output of the pwd command shows that user is in the project directory, which is in the /home/dsahu/ directory.
  b. Open and edit the bash profile using your favorite text editor
    >nano ∼/.bash_profile
  c. Add the output of the pwd command to the last line.
    >export protocol_dir="/home/dsahu/project"
  d. Press ‘control’ + ‘x’ key to exit. You will be prompted to save the file. Press ‘Y’ for Yes to save the changes and ‘enter’.
  e. Activate the changes in the bash_profile
    >source ∼/.bash_profile

### Set executable permission to all the scripts

**Timing: 1 min**

3. Navigate to STAR_protocols_GV_calling directory.

>cd STAR_protocols_GV_calling/

4. Navigate to ‘scripts’ directory and set executable permission to all the scripts executable using the following command

>cd scripts/

>chmod +x ∗

### Software requirement

This protocol describes a computational approach that is based on several Linux-based software including gdc-client, SAMtools (Li et al., 2009), BCFtools (Danecek et al., 2021; Li, 2011a), VarDictJava (Lai et al., 2016) and numerous packages available in R. See Key resources table and go through the provided link for software installation in Linux. To make software executable from anywhere in the Linux system, user needs to add the path of the software to the $PATH environmental variable. For R and required R packages see Materials and equipment.

### Download datasets

**Timing: 20 min**

This protocol requires whole-exome-sequences from the normal samples (wxs-normal), whole-exome-sequences from the tumor samples (wxs-tumor) and RNA-sequences from the tumor samples (rnaseq-tumor) from The Cancer Genome Atlas (TCGA) patients available in the Genomics Data Commons (GDC) data portal (Grossman et al., 2016). The aligned reads in the Binary Alignment Map (BAM) format should be downloaded from the database of Genotypes and Phenotypes (dbGaP), target genes genomic regions in the Browser Extensible Data (BED) format should be downloaded from the GENCODE v37 (Frankish et al., 2019) database, and the reference human genome sequence (hg38) in the FASTA format should be downloaded from the GDC. We demonstrate the use of the pipeline on 5 wxs-normal samples, 7 wxs-tumor samples and 7 rnaseq-tumor samples. The BAMs used in this protocol were from the TCGA lower grade glioma (LGG) cohort (Brat et al., 2015). The names of test datasets are provided in the GitHub repository above.

5. Navigate to ‘data’ directory with three subdirectories ‘BAM’, ‘reference_data’ and ‘samples’. The reference_data directory contains gene annotation BED file from the GENCODE database. Download GDC reference genome (hg38) FASTA sequence (GRCh38.d1.vd1.fa.tar.gz) from https://gdc.cancer.gov/about-data/gdc-data-processing/gdc-reference-files/.
  a. Unzip and place the reference human genome FASTA sequence in the ‘/data/reference_data/HSapiens/hg38/’ directory.
    >cd data/reference_data/HSapiens/hg38/
    >tar xvzf GRCh38.d1.vd1.fa.tar.gz
  b. SAMtools should be loaded in the system PATH. We used the command below to load samtools in our online Linux cluster.
    >module load samtools/1.12
  c. Index the FASTA sequence.
    >samtools faidx GRCh38.d1.vd1.fa

6. Navigate to ‘BAM’ directory and download BAM files for wxs-normal samples, wxs-tumor samples, and rnaseq-tumor samples from the provided manifest files and place them in their respective directory. A GDC token is required to download BAMs from the GDC database.
  a. Download BAM files using the following command

>cd STAR_protocols_GV_calling/data/BAM/wxs-normal/

>gdc-client download -m gdc_manifest_wxs-normal.txt -t token.txt

>cd STAR_protocols_GV_calling/data/BAM/wxs-tumor/

>gdc-client download -m gdc_manifest_wxs-tumor.txt -t token.txt

>cd STAR_protocols_GV_calling/data/BAM/rnaseq-tumor/

>gdc-client download -m gdc_manifest_rnaseq-tumor.txt -t token.txt

### Create input list

**Timing: 1 min**

7. Variant calling using VarDictJava requires an input.list that contain path to folders of BAM files. Prepare an input list for wxs-normal, wxs-tumor and rnaseq-tumor data types, separately.
  a. Run the following command from the directory that contains only folders of BAM files and do not contain other information except previous logs. For example, let us create an input.list for wxs-normal BAMs.
    >cd data/BAM/wxs-normal/
    >find $(pwd) -type d | awk '!/logs/' | sed '1d' > input.list
  b. Rename the input.list as you wish.
    >mv input.list wxs-normal_input.list

### Output directory

8. The default output for this protocol is within the following directory.

>cd STAR_protocols_GV_calling/analysis/

## Key resources table

**Table:** 

REAGENT or RESOURCE	SOURCE	IDENTIFIER
**Deposited data**		

TCGA-LGG aligned reads wxs (IlluminaHiSeq data, controlled access)	GDC and dbGaP	TCGA-LGG-WXS
TCGA-LGG aligned reads rnaseq (IlluminaHiSeq data, controlled access)	GDC and dbGAP	TCGA-LGG-RNAseq
TCGA-LGG curated survival data	(Ceccarelli et al., 2016) and cBioPortal (Cerami et al., 2012; Gao et al., 2013)	https://www.ncbi.nlm.nih.gov/pmc/articles/PMC4754110/bin/NIHMS746836-supplement-7.docx; https://www.cbioportal.org/study/clinicalData?id=lgg_tcga
Data and analyses	This paper	dbGaP database; accession: phs000178.v11.p8; https://www.ncbi.nlm.nih.gov/projects/gap/cgi-bin/study.cgi?study_id=phs000178.v11.p8
Code	This paper	GitHub: https://github.com/ds21uab/STAR_protocols_GV_calling

**Software and algorithms**		

VarDictJava (v1.8.2)	(Lai et al., 2016)	https://github.com/AstraZeneca-NGS/VarDictJava
SAMtools (v1.12)	(Li et al., 2009)	http://www.htslib.org/download/
BCFtools (v1.9)	(Danecek et al., 2021; Li, 2011a)	http://www.htslib.org/download/
Tabix (v0.2.6)	(Li, 2011b)	http://www.htslib.org/doc/tabix.html
Annovar	(Wang et al., 2010)	https://annovar.openbioinformatics.org/en/latest/user-guide/gene/
GnomAD (v3.1.1) whole genome sequencing	(Karczewski et al., 2020)	https://gnomad.broadinstitute.org/
R	(RCoreTeam, 2021)	https://www.r-project.org/
RStudio	(RStudioTeam, 2020)	https://www.rstudio.com/
GDC Data Transfer Tool	GDC	https://gdc.cancer.gov/access-data/gdc-data-transfer-tool
Plink (v1.90b6.16)	(Chang et al., 2015; Purcell et al., 2007)	https://www.cog-genomics.org/plink/1.9/

## Materials and equipment

R software and required R packages: While newer versions of some of these packages are available, this protocol was developed with R v4.0.3, RStudio v4.1.1, and the following versions of R packages:

• data.table (v.1.14.0)
• dplyr (v1.0.6)
• forcats (v0.5.1)
• stringr (v1.4.0)
• purrr (v0.3.4)
• readr (v1.4.0)
• tidyr (v1.1.3)
• tibble (v3.1.2)
• ggplot2 (v3.3.3)
• Tidyverse (v.1.3.1)
• survival (v3.2-13) (Therneau, 2021)

Hardware Recommendations:

• Operating system: GNU/Linux
• Memory: 100 GB (memory requirement depends on size of the dataset)
• Processors: 1 required, 5 recommended

The script in this protocol follows Simple Linux Utility for Resource Management (SLURM)-based schema and requires submission of the job to an online Linux cluster. The steps discussed below loop over each BAM file or the downstream output files that were generated. Therefore, it is easier to submit similar jobs as job arrays, which will create a number of independent jobs (corresponding to the defined number of tasks) and execute them simultaneously. It is common to load pre-installed software as an environmental module available on the Linux cluster. User needs to load the relevant modules on their cluster. This protocol expects that R, VarDictJava, SAMtools and BCFtools are installed on your local or online Linux cluster and loaded in the system PATH.

## Step-by-step method details

A typical workflow to call germline variants from the wxs-normal samples, wxs-tumor samples, and rnaseq-tumor samples (the three ‘data types’ in this protocol) requires six parts. The first part is variant calling using VarDictJava. The second part is the preprocessing of VCFs and extraction of genotype status of each variant for each sample. We then perform union of all the unique variants from all three data types. The third part is to calculate the sequencing coverage of the union of unique variants. The fourth part is to merge genotype status file and sequencing coverage file of each sample to determine the status of each variant after correction for low sequence coverage. The variant status at positions with fewer than ten reads for a given sample is changed to unknown. The fifth part is to combine variant status file from each sample to create a large multi-sample VCF file. Finally, in the sixth part, at positions at which variant status is listed as unknown in the wxs-normal samples (because of low sequence coverage) we will insert variant calls made from the corresponding wxs-tumor samples. If the variant status is still unknown in a normal sample but is called in the corresponding rnaseq-tumor sample, then the rnaseq-derived variant is inserted to create the final combined wxs-rnaseq variant call set.

***Note:*** Run parts 1-5 separately for wxs-normal samples, wxs-tumor samples, and rnaseq-tumor samples.

### Part 1: Variant calling using VarDictJava

**Timing: 2 h (computational time scales with number of samples and available resources)**

**Figure 1:**
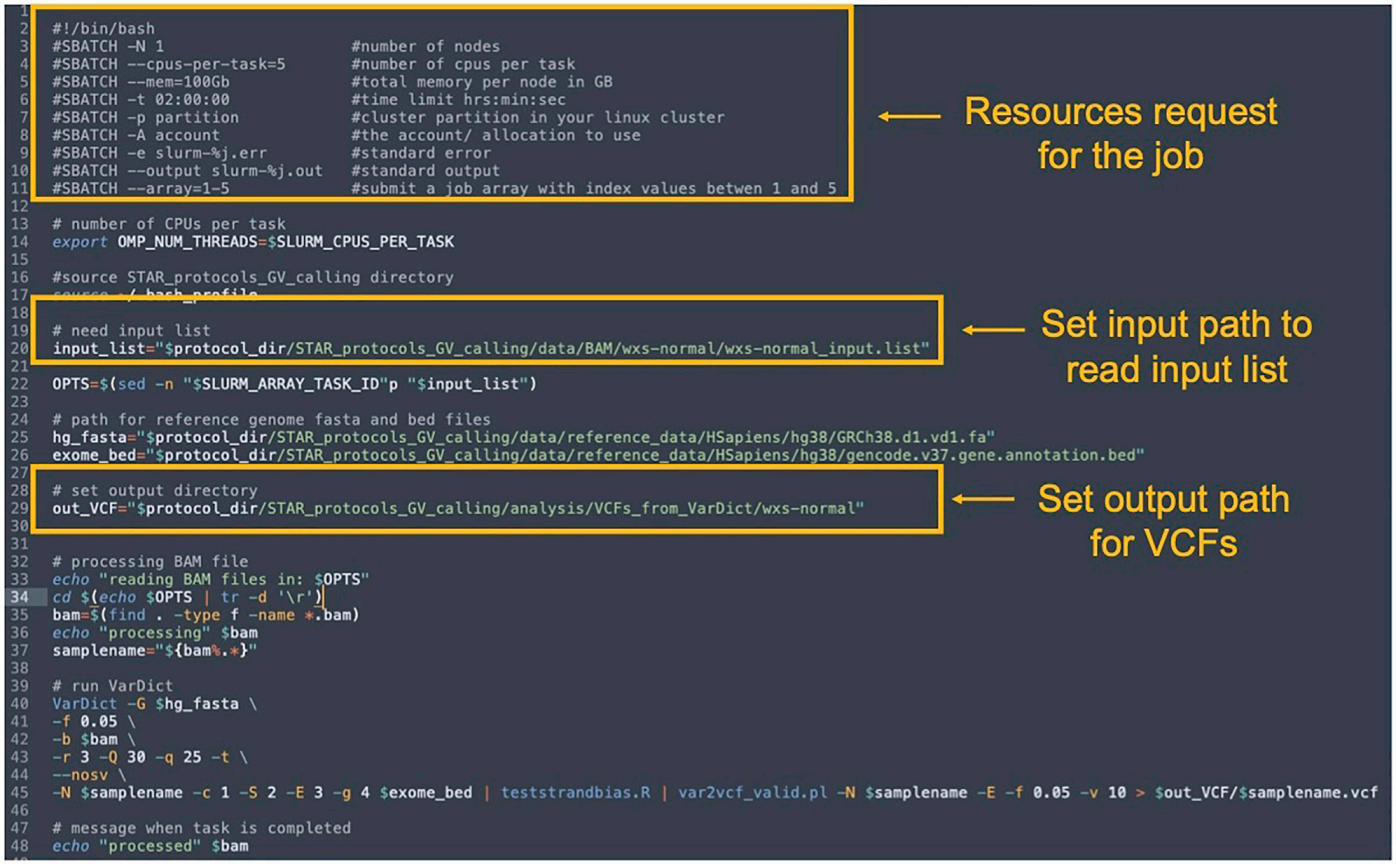
Example showing the structure of the VariantCallingFrom_VarDict.sh bash script The highlighted text in yellow demonstrates resources requested for the job, where to set path to read input.list, and path to output VCF files. To open and edit bash script use any text editor like nano.
  d. The default output for this step is stored in the following directory.
    >cd STAR_protocols_GV_calling/analysis/VCFs_from_VarDict/
  e. User should create directory ‘wxs-normal’, ‘wxs-tumor’, and ‘rnaseq-tumor’ in the ‘VCFs_from_VarDict’ directory to store VCFs for respective data types.
    >mkdir wxs-normal
    >mkdir wxs-tumor
    >mkdir rnaseq-tumor
  f. Set output path in the script accordingly (See Figure 1). For example, output path for VCFs for wxs-normal samples can be set as shown below
    >out_VCF="$protocol_dir/STAR_protocols_GV_calling/analysis/VCFs_fr
     om_VarDict/wxs-normal"
  g. Run script using the following command
    >sbatch
    $protocol_dir/STAR_protocols_GV_calling/scripts/VariantCallingFrom
    _VarDict.sh
  h. If the user online Linux cluster supports Terascale Open-source Resource and QUEue Manager (TORQUE) system,
    i. Change the structure of the job script in the VariantCallingFrom_VarDict.sh as described below
      >#PBS -k o ##keep the job output
      >#PBS -N JobName ## name of the job
      >#PBS -l nodes=1 ##number of nodes
      >#PBS -l ncpus=5 ## number of cpus
      >#PBS -l mem=100Gb ## memory requested per node in Gb
      >#PBS -l walltime=02:00:00 ## time limit hrs:min:sec
      >#PBS -q queue ##partition requested in the cluster
      >#PBS -e torque-%j.err ##standard error
      >#PBS -o torque-%j.out ##standard output
      >#PBS -t 1-5 ##number of array jobs is from 1 to 5
    ii. Run script using the following command
      >qsub
      $protocol_dir/STAR_protocols_GV_calling/scripts/VariantCallingFrom
      _VarDict.sh

1. Variant calling on wxs-normal BAMs, wxs-tumor BAMs, and rnaseq-tumor BAMs using VarDictJava. The settings were set as default except for requiring mapping quality greater than 30, base quality greater than 25, a minimum of 3 reads supporting a variant, minimum allele frequency of 5%, no structural variant calling, and the removal of duplicate reads.
  a. Navigate to ‘scripts’ directory and open VariantCallingFrom_VarDict.sh script in your favorite text editor.
    >nano VariantCallingFrom_VarDict.sh
  b. As the script in this protocol follows SLURM based schema. The structure of the job script is described below.
    >#SBATCH -N 1 ##number of nodes
    >#SBATCH --cpus-per-task=5 ##number of cpus per task
    >#SBATCH -mem=100Gb ##memory requested per node in GB
    >#SBATCH -t 02:00:00 ##time limit hrs:min:sec
    >#SBATCH -p partition ##partition requested in the cluster
    >#SBATCH -A account ## account to be charged
    >#SBATCH -e slurm-%j.err ##standard error
    >#SBATCH -output slurm-%j.out ##standard output
    >#SBATCH -array=1-5 ##number of array jobs is from 1 to 5
    ***Note:*** Set the number of nodes, number of cpus, requested memory, and time in the job script according to the number of samples to be processed and resources available on the user online Linux cluster. The numbers designating the array jobs correspond to the numbers of the jobs in the input list.
  c. As different data types are processed, set the path in the script to the input list that corresponds to the data type (See Figure 1). For example, to set path of input.list for wxs-normal BAMs
    >input_list="$protocol_dir/STAR_protocols_GV_calling/data/BAM/wxs
     -normal/wxs-normal_input.list"

### Part 2: Preprocessing of VCF files

**Timing: 30 min**

2. Index VCF file and extract variants that received a PASS (shown in the “filter” column) in the indexed VCF file.
  a. The default output for this step is stored in the following directory.
    >cd STAR_protocols_GV_calling/analysis/PASS_variants/
  b. User should create directory for each data type in the ‘PASS_variants’ directory to store outputs for respective data type.
    >mkdir wxs-normal
    >mkdir wxs-tumor
    >mkdir rnaseq-tumor
  c. Set output path in the extract_pass_variants.sh script for each data type accordingly. For example, let’s set the output path to store indexed passed variants for wxs-normal samples
    >nano extract_pass_variants.sh
    >out_VCF="$protocol_dir/STAR_protocols_GV_calling/analysis/PASS_va
     riants/wxs-normal"
  d. Run the script from the directory where VCFs from VarDictJava are located.
    >$protocol_dir/STAR_protocols_GV_calling/scripts/extract_pass_varia
    nts.sh

3. Remove header section from VCFs. Run the script from the directory where indexed pass variants are located.

>$protocol_dir/STAR_protocols_GV_calling/scripts/remove_header_VCF

.sh

4. Extract unique variants from all the passed variants VCF files. Run script from the directory where VCF files without header are located. This script will output allVCF_variants.txt file (total variants from all the passed variants VCFs), and unique_variants.txt file (unique variants from all the passed variants VCFs). User may rename the unique_variants.txt for each data type accordingly.

>$protocol_dir/STAR_protocols_GV_calling/scripts/extract_uniqueVar

iants.sh

***Note:*** This bash script extract_uniqueVariants.sh includes R script extract_uniqueVariants.R. See ‘Potential problem 3’ to solve issue with cannot find the R script.

5. Extract genotype status for each variant from each sample. Here, we extract chromosome, position, reference allele, observed alternate allele, mutation status, total depth, and variant depth of each variant from each VCF file.
  a. The default output for this step is in the following directory.
    >cd STAR_protocols_GV_calling/analysis/genotype_status/
  b. User should create separate directory in the ‘genotype_status’ directory to store outputs for each data types.
    >mkdir wxs-normal
    >mkdir wxs-tumor
    >mkdir rnaseq-tumor
  c. Set output path in the extract_genotype_from_passedVariants.sh script for each data type. For example, set output path for wxs-normal samples
    >nano extract_genotype_from_passedVariants.sh
    >out_VCF="$protocol_dir/STAR_protocols_GV_calling/analysis/genotyp
     e_status/wxs-normal"
  d. Run script from the indexed passed variants VCFs directory
    >$protocol_dir/STAR_protocols_GV_calling/scripts/extract_genotype_
    from_passedVariants.sh

6. Merge unique variants obtained from the wxs-normal, wxs-tumor and rnaseq-tumor data type by chromosome, position, reference allele and observed alternate allele. Here, we will keep the union of unique variants obtained from all the three data types and save them as union_wxs_rnaseq_variants.bed file. User can use R to merge the unique variants obtained from the three data types
  a. Load required R package
    >library (data.table)
  b. Load data in R
    >wxs_normal=fread("/STAR_protocols_GV_calling/analysis/PASS_varian
       ts/wxs-normal/unique_variants.txt",sep="∖t",
       check.names=FALSE, header=TRUE)
    >wxs_tumor=fread("/STAR_protocols_GV_calling/analysis/PASS_variant
       s/wxs-tumor/unique_variants.txt",sep="∖t",
       check.names=FALSE, header=TRUE)
    >rnaseq_tumor=fread("/STAR_protocols_GV_calling/analysis/PASS_vari
       ants/rnaseq-tumor/unique_variants.txt",sep="∖t",
       check.names=FALSE, header=TRUE)
  c. Convert data.table to data.frame
    >wxs_normal <- setDF (wxs_normal)
    >wxs_tumor <- setDF (wxs_tumor)
    >rnaseq_tumor <- setDF (rnaseq_tumor)
  d. Convert into list
    >variant_list = list(wxs_normal, wxs_tumor, rnaseq_tumor)
  e. Merge list based on chromosome, position, reference allele, and alternate allele
    >union_variants= Reduce(function(x,y) merge(x, y, by.x = c("CHROM",
       "POS", "REF", "ALT"), by.y = c("CHROM", "POS",
       "REF", "ALT"), all.x=TRUE, all.y=TRUE),
      variant_list)
  f. Drop unwanted columns
    >drop <- c("REF", "ALT")
    >union_variants<-union_variants[,!colnames(union_variants) %in%
      drop]
  g. Save union variants in the analysis directory
    >fwrite(union_variants,"/STAR_protocols_GV_calling/analysis/union_
    wxs_rnaseq_variants.txt",col.names=FALSE,quote=FALSE,row.names=FAL
    SE,sep="∖t")
  h. Terminate the current R session. User will be prompted to save the workspace. Please type ‘no’ if you wish not to save the workspace.
    >quit()
  i. Now we are outside R. Convert the union_wxs_rnaseq_variants.txt file into .bed file in the Linux command line
    >mv union_wxs_rnaseq_variants.txt union_wxs_rnaseq_variants.bed

### Part 3: Calculate sequencing coverage of union variants

**Timing: 2 h**

7. In this step, we will calculate the sequencing coverage of union variants (listed in the union_wxs_rnaseq_variants.bed file) on all BAMs requiring mapping quality greater than 30.
  a. The default output for this step is in the following directory.
    >cd STAR_protocols_GV_calling/analysis/variant_coverage
  b. User should create separate directory for each data type in the ‘variant_coverage’ directory to store output for each data type.
    >mkdir wxs-normal
    >mkdir wxs-tumor
    >mkdir rnaseq-tumor
  c. Set output path in the script accordingly. For example, set output path for wxs-normal samples
    >nano UnionVariantCoverageFrom_Samtools_depth.sh
    >out_depth="$protocol_dir/STAR_protocols_GV_calling/analysis/varia
       nt_coverage/wxs-normal"
  d. Run the script using the following command
    >sbatch
    $protocol_dir/STAR_protocols_GV_calling/scripts/UnionVariantCovera
    geFrom_Samtools_depth.sh
    **CRITICAL:** union_wxs_rnaseq_variants.bed file and input.list that contain the location of the BAM files are required as input files. Set path of input files in the script. Set array jobs with number of jobs in the input list.

### Part 4: Determine status of each variant in each patient for each data type

**Timing: 5 min**

**Figure 2:**
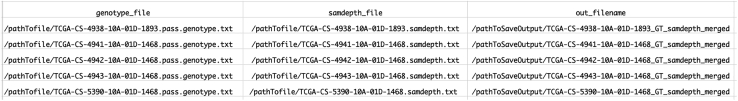
Example showing how to prepare the input_genotype_samdepth.txt file The first column is the path where the genotype status of each sample is located, the second column is the path of sequencing coverage of each sample is located, and the third column describes the path where to save the variant status file.
  b. The default output for this step is in the following directory.
    >cd STAR_protocols_GV_calling/analysis/variant_status
  c. User should create separate directory for each data type in the ‘variant_status’ directory to save output for each data type.
    >mkdir wxs-normal
    >mkdir wxs-tumor
    >mkdir rnaseq-tumor
  d. Set path in the out_filename column in the input_genotype_samdepth.txt file to save results for each data type. For example, set output path for wxs-normal samples
    >STAR_protocols_GV_calling/analysis/variant_status/wxs-normal/
  e. Place the input_genotype_samdepth.txt file in the analysis directory.
  f. Run the script using the following commands from the analysis folder.
    >sbatch
    $protocol_dir/STAR_protocols_GV_calling/scripts/determineVariantSt
    atus.sh
    ***Note:*** The bash script determineVariantStatus.sh includes R script determineVariantStatus.R. Set array jobs with number of jobs in the input list.

8. Determine status of each variant using the genotype status and sequencing coverage of each variant in each sample. The variant status at positions fewer than ten reads for a given patient is changed to unknown. If the sequencing coverage of the variant is more than ten reads and no alternate allele is reported for that variant, then the status of that given variant is changed to Homozygous reference.
  a. Input files: prepare a tab separated input_genotype_samdepth.txt file as shown in Figure 2 with three columns that describe ‘genotype_file’ (the location of the genotype status file with filename), ‘samdepth_file’ (the location of the union variants sequencing coverage file with filename), and ‘out_filename’ (the location of the output file with filename) for each sample. For the output file filename add _GT_samdepth_merged suffix at the end of the filename.

### Part 5: Creating multi-sampled variant status file

**Timing: 1 h**

9. Combine variant status file of each sample to create a multi-sample VCF which stores the variant status from all the samples.
  a. The default output for this step is in the following directory.
    >cd STAR_protocols_GV_calling/analysis/combined_variant_status
  b. User should create a separate directory in the ‘combined_variant_status’ to save output for each data type.
    >mkdir wxs-normal
    >mkdir wxs-tumor
    >mkdir rnaseq-tumor
  c. Run the script using the following commands
    >sbatch
    $protocol_dir/STAR_protocols_GV_calling/scripts/combineAllSamplesV
    ariantStatus.sh
    **CRITICAL:** The bash script combineAllSamplesVariantStatus.sh includes the R script combineAllSamplesVariantStatus.R. In the R script set full path for input files (i.e., variant status file obtained from the previous step) and the output path where combined variants from all samples to be saved. The memory and time may vary based on the number of samples to be processed.

10. The script above outputs a large table which contains variant status from all the samples. However, we need to do a bit of legwork to get our data into reasonable shape. First, the combined variants status file contains millions of variants, and it will take too much of memory and time for data pre-processing. Therefore, we will first split the large-combined variant status file into chunks of small files keeping the same number of columns but split based on fixed number of rows. In this file, each line corresponds to one variant.
  >file=combinedVariantStatusFromAllSamples.txt
  >head -1 $file > header.txt
  >sed '1d' $file > combined_variants_without_header.txt
  >split -d -l 50000 combined_variants_without_header.txt -a 4 –
   additional-suffix=.txt segment_
  With the above commands we did the following:
  a. -d #add numeric suffix to split file
  b. -l #number of lines in each of the smaller files
  c. -a #suffix length
  d. --additional-suffix
  e. segment_ #split file prefix

11. Prepare input list for pre-processing

>find $(pwd) -type f -name "segment∗" > input.txt

>sort -V input.txt > input.list

12. The pre-processing step takes each split file from the input.list and first removes the unwanted columns. Then for each variant it extracts the chromosome, base-position, the reference allele, the observed alternate allele and the variant status for each sample. From the fifth column onwards, the column names change to the respective sample names from TCGA. Modify the script if you have different sample names.
  a. Input files: input.list and header.txt file
  b. Output files: processed combined variant status file for each split file
  c. Run script from the folder where input.list and header.txt is stored
    >sbatch
    $protocol_dir/STAR_protocols_GV_calling/scripts/processCombinedVar
    iants.sh
    ***Note:*** The bash script processCombinedVariants.sh includes R script processCombinedVariants.R. Set array jobs with number of jobs in the input list.

13. Once preprocessing of each split file is finished, the next step is to merge them. Run the command below from the command line interface

>awk 'NR == 1 || FNR > 1' processed_CombinedVariants_∗.txt >

processed_CombinedVariantsFromAllSamples.txt

14. Remove variants which are either unknown or Homozygous reference across all samples from the processed_CombinedVariantsFromAllSamples.txt file. Run the script below from the directory where the processed_CombinedVariantsFromAllSamples.txt file is located. The script will output the processed_CombinedPotentialSNPs.txt file.

>Rscript

$protocol_dir/STAR_protocols_GV_calling/scripts/extractPotentialSN

PsFromCombinedVariants.R

### Part 6: Insert variant call for unknown variant status in normal samples

**Timing: 40 min**

15. The goal of this step is to insert variant status at positions listed as unknown in the wxs-normal sample from the corresponding tumor sample. If the variant status is still unknown in the normal sample, then we will insert variant status of the rnaseq-derived variant from the corresponding rnaseq-tumor sample. This step will allow us to create a final combined wxs-rnaseq variant call set.
  a. Input files:
    i. processed_CombinedPotentialSNPs.txt file obtained for wxs-normal, wxs-tumor and rnaseq-tumor data types.
    ii. Patient list with Case_ID and Sample_Barcode. If a matched tumor /normal sample have whole exome sequencing and associated RNA sequencing file, then the different datasets from the patient will have the same Case_ID. Prepare separate patient list for wxs-normal, wxs-tumor and rnaseq tumor samples. Set the column names of each file as suggested:
       wxs-normal: Case_ID and normal
       wxs-tumor: Case_ID and tumor
       rnaseq-tumor: Case_ID and rnaseq
  b. Patient list for this protocol is provided in the sample directory and can be accessed by the following command
    >cd STAR_protocols_GV_calling/data/samples
  c. Run the script using the following command. Run the script from the analysis folder.
    >sbatch
    $protocol_dir/STAR_protocols_GV_calling/scripts/fillUnknownVariant
    sInNormalSamples.sh
    **CRITICAL:** The bash script fillUnknownVariantInNormalSamples.sh includes R script fillUnknownVariantInNormalSamples.R. Set full path for the patient files and the processed_CombinedPotentialSNPs.txt file in the script.
  d. The output from step six includes four files
    i. combined_normal_unknown_filled.txt
    ii. combined_normal_unknown_filled_arranged.txt
    iii. final_merged_wxs_rnaseq_variants.txt (stores the combined set of variant calls from the wxs-normal, wxs-tumor and rnaseq-tumor samples)
    iv. final_variantsForAnnovar.txt (combined set of variant call formatted as input file for variant annotation using Annovar)

### Part 7: Examples of downstream analyses

In the previous steps, we have identified germline variants from the whole-exome sequences and rna-sequences in the TCGA-LGG cohort. Several downstream analyses can be performed to evaluate the quality and clinical relevance of the identified germline variants.

16. Use Annovar software to perform functional annotation of germline variants such as in which gene the variant is located, whether variant is in the exonic, intronic, UTR, promoter, or splicing region of a gene or is in the intergenic region.
17. Use Annovar to determine the allele frequencies of germline variants in whole-genome sequencing data from various ethnic populations such as listed in the GnomAD database.
18. Calculate the allele frequency of the germline variant in this study using the following formula:

$$Allele\;frequency=\frac{\left(2\text{∗}\#HA\right)+\#HT}{\left(2\text{∗}\#HR\right)+\left(2\text{∗}\#HA\right)+\left(2\text{∗}\#HT\right)}$$

where #HR is the total number of patients with Homozygous reference allele, #HT is the total number of patients with Heterozygous allele, and #HA is the total number of patients with Homozygous alternate allele.

**Figure 3:**
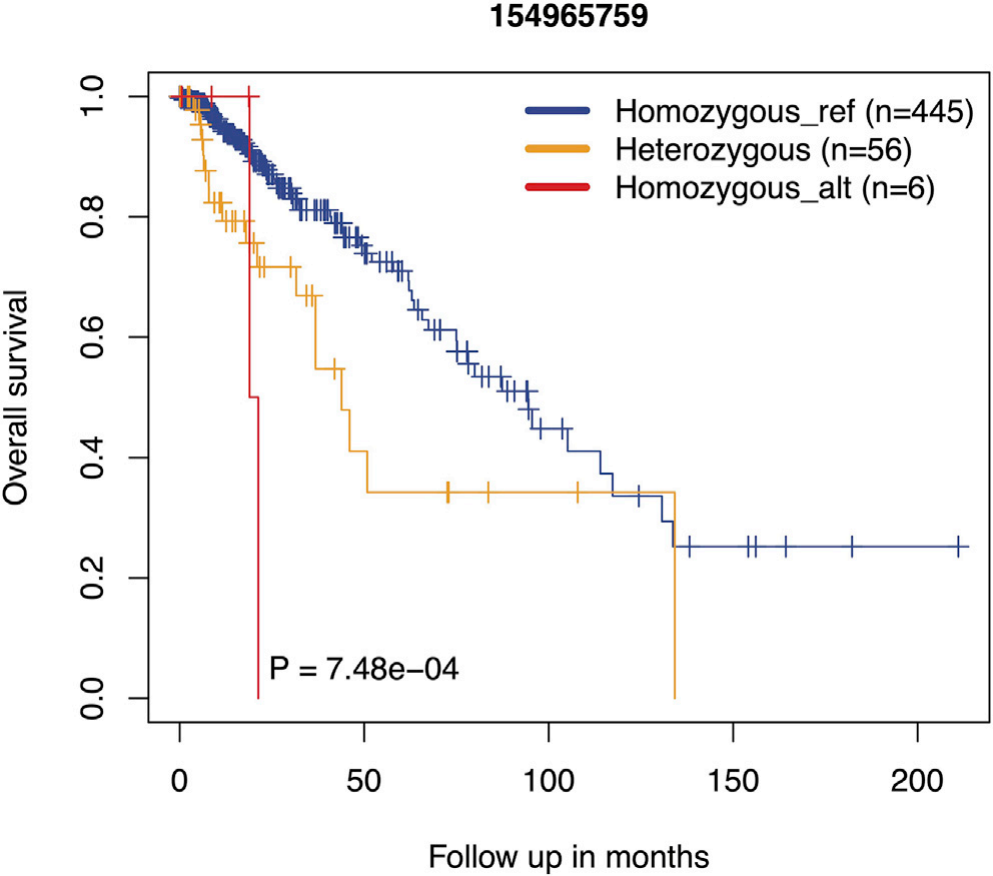
Survival estimates of overall survival in TCGA lower-grade glioma patients Kaplan-Meier plot shows survival probability of lower grade glioma patients with Homozygous-reference, Heterozygous and Homozygous alternate genotype for variant rs1131397. This variant is located on chromosome 1 at 154965759 genomic locus, where the reference base G is changed to C. The p-value was obtained by log-rank (Mantel-Haenszel) test. Here, P indicates p value.
  g. Multivariate Cox regression analysis (Figure 4): Convert the confounding clinical risk factors into ordinal scale
    i. Age
    ii. Percent_aneuploidy
    iii. Histology: oligodendroglioma = 1; oligoastrocytoma = 2; astrocytoma = 3
    iv. Grade: G2 = 1; G3 = 2
    v. IDH_status: Mutant = 1; WT = 2
    vi. Mutation_count
    vii. Chr1p/19q_codeletion: codel = 1; non-codel = 2
    viii. MGMT_promoter_status: Methylated = 1; Unmethylated = 2
    ix. Chr7gain/Chr10loss: No combined CNA = 1; Gain chr 7 & loss chr 10 = 2
    x. Treatment_site: Other = 1; Duke = 2; Henry Ford Hospital = 3
    xi. Principal component 3 (PC3)
      >variant.cox <- coxph(Surv(OS_months, OS_status) ∼ labels + Age +
       Percent_aneuploidy + Histology + Grade +
       IDH_status + Mutation_count + Chr7gainORChr10loss
       + MGMT_promoter_status + Chr1OR19q_codeletion +
       Treatment_site + PC3, data=data)
      >summary(variant.cox)

19. Calculate the correlation of the allele frequencies in the four variant call sets (final merged variant call set, wxs-normal, wxs-tumor, rnaseq-tumor datasets) with each other and with the allele frequency from GnomAD using GGally R package. In our study, the allele frequencies in the combined data set from ∼500 patients gave a correlation coefficient of >0.9 with the allele frequencies in GnomAD.
20. Determine whether the germline variant calls separate patients based on self-reported race using the PLINK software.
21. Determine the genetic linkage between germline variants at different loci by performing Linkage Disequilibrium analysis.

22. Determine for a given germline variant whether there is a significant difference in survival outcome in the minor allele compared to the reference major allele (Chatrath et al., 2019; Chatrath et al., 2020). Here, we have compared the overall survival outcome between LGG patients (n=507) with different rs1131397 (Chr1-154965759-G-C) genotypes. See Expected outcomes for the discussion of the obtained results.
  a. Load required R package
    >library(survival)
  b. Variant genotype is encoded as character vector. Model genotype in ordinal scale
    i. Homozygous_ref: 0
    ii. Heterozygous: 1
    iii. Homozygous_alt: 2
  c. Save variant genotype (converted in ordinal scale) for each patient
    >labels <- variant_genotype
  d. Download the survival data to the working directory (see Key resources table) and load it with the following command
    >data <- read.csv("survivalData_from_Ceccarelli_LGG.csv",
      stringsAsFactors=FALSE, strip.white=TRUE,
      check.names=FALSE, header=TRUE, fill=TRUE)
  e. Survival analysis
    >Risk_survival= survfit(Surv(OS_months, OS_status)∼ labels,
      data=data)
    >pvalue_Risk_survival= survdiff(Surv(OS_months, OS_status)∼
       labels, data=data, rho=0)
    >p.val= 1 - pchisq(pvalue_Risk_survival$chisq,
     length(pvalue_Risk_survival$n) - 1)
  f. Plot the Kaplan-Meier curves (Figure 3)
    >par(mar=c(5,6,3,2), cex.axis=1, cex.lab=1, cex.main=1,
    font.axis=1, font.lab=1, par(font=1))
    >plot(Risk_survival, lty=c(1,1), col=c("blue", "orange", "red"),
    mark.time=TRUE, xlab="Follow up in months", ylab="Overall
    survival", lwd=1)
    >text(50, 0.05, paste0("P =", " ", format(p.val, scientific=TRUE,
    digits=3)))

**Figure 4:**
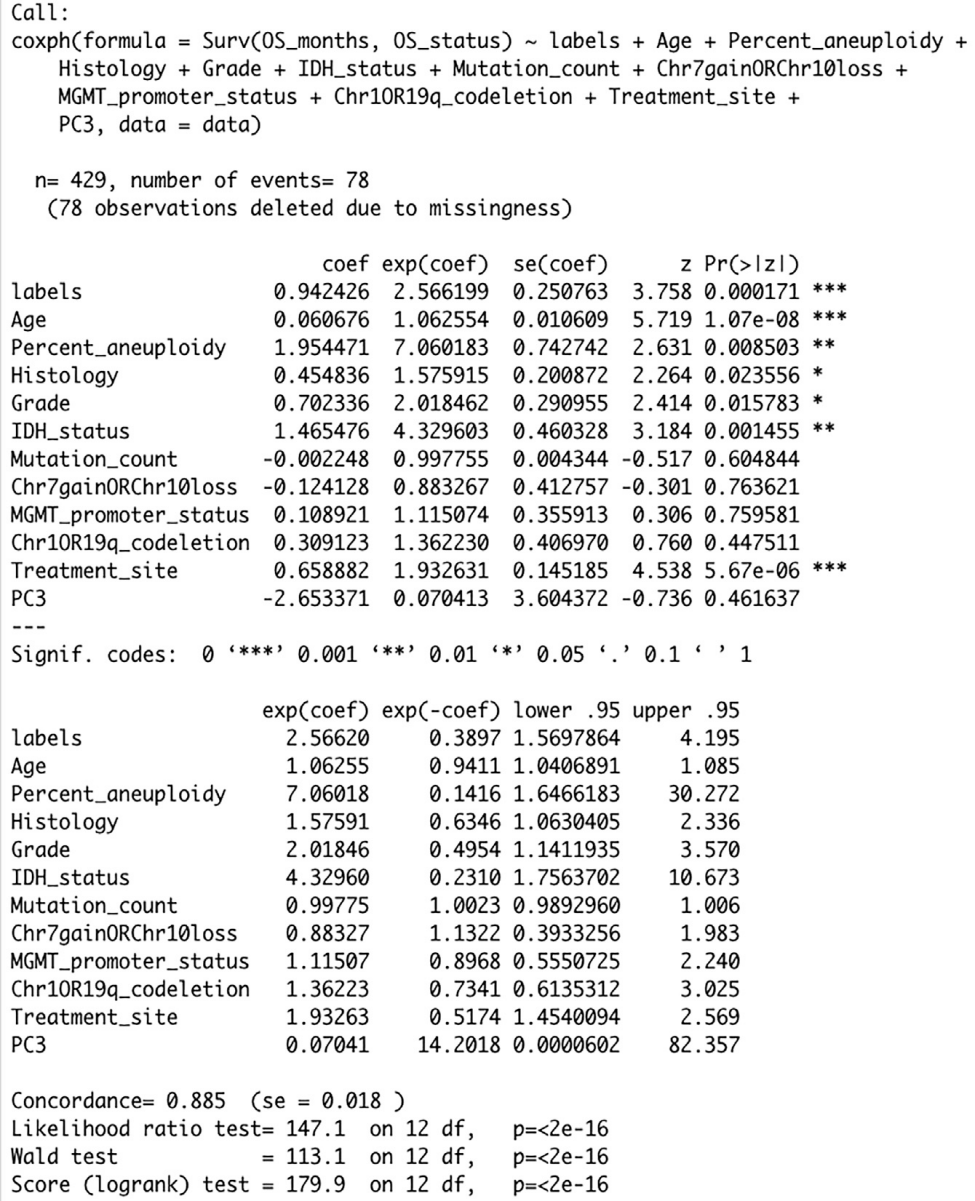
Multivariable Cox regression analysis Cox regression analysis shows that lower grade glioma patients with minor allele of variant rs1131397 is associated with poor outcome. The significant p-value for the labels variable indicate that minor allele is an independent predictor of overall survival after adjusting for the confounding clinical risk factors. Here, Pr(>|z|) indicates p value.

23. The output for multivariate Cox regression analysis includes coefficient, hazard ratio, z-score and p value for each variant. As multiple variants are tested, it is important to correct the p values after Cox regression. In our study, 196,022 variants were tested. False discovery was performed through Bonferroni Correction.
24. Determine whether a given germline variant is predictive of increase or decrease in tumor mutation burden and responsiveness to immune checkpoint chemotherapy (Chatrath et al., 2021a).
25. For germline variants that are significantly associated with a phenotype (increase or decrease of survival, tumor mutation burden, responsiveness to specific therapy), determine whether the variant is associated with differences in gene expression of nearby genes by performing expression quantitative trait loci (eQTLs) analysis. For more details on other downstream analyses that can be performed with germline variants, please refer to (Chatrath et al., 2021b).

## Expected outcomes

This protocol presents instructions to integrate the whole-exome sequencing datasets from normal and tumor samples and RNA sequencing dataset from the tumor samples of the TCGA-LGG cohort to determine the germline variants.

Germline variants from the TCGA datasets are typically called in wxs-normal blood samples. This approach captures exonic region of the genome, allowing one to find the variants within genes. However, sequence (and variants) outside the exonic region, such as the promoters, introns, intron-exon boundaries and UTRs are also captured as a byproduct of whole-exome capture and are present in wxs data (Guo et al., 2012; Samuels et al., 2013). In this protocol we combine the information from wxs-normal, wxs-tumor and rnaseq-tumor datasets for a given patient to get additional sequence coverage at a base and thus call germline variants that may not have had sufficient sequence depth in the wxs-normal data. In addition, this approach can be used to call variants in patient sets where wxs-normal is not available. Tumor-specific mutation and RNA editing changes are also called as variant in wxs-tumor and rnaseq-tumor, but these are filtered out at a later stage by focusing only on variants that are known to exist in the general population with a minor allele frequency ≥ 0.05. Lower thresholds for minor allele frequency can be used when more patient datasets are available.

There were some choices we made in our protocol that may be changed by a user. We use a minimal sequencing coverage threshold of 10 before a variant status is called because that gives us enough confidence that if a variant was present at that locus, it should have been sampled by then (and sampled at least 3 times). This is an arbitrary threshold and other users can set other thresholds if they so desire, but one has to balance the need for finding sufficient patients with a minor allele with the need to be 100% confident that the variant call is correct. In addition, we have a filter that a minimum of three reads should support the reported variant, and this too can be changed by the user. Note that we also removed reads that may have come from PCR duplicates, so that only one read with the identical start position is kept among all the duplicates. Finally, we turned off the calling of structural variants in this analysis so that no insertions, deletions, duplications, inversions, or large copy number variations were called.

Our method has the benefit of determining the genotype status of a variant with low sequencing coverage in wxs-normal sample, by taking the genotype status of that variant from the corresponding wxs-tumor sample, where the variant at that position may be supported with higher sequencing coverage. However, if we are still unable to determine the genotype status, we insert the genotype status from the rnaseq counterparts. This method has not significantly affected the accuracy of the variant call in our study because the allele frequency calculated from the rnaseq tumor datasets were significantly positively correlated (r > 0.98) with the allele frequencies from the wxs-normal, wxs-tumor and combined-wxs-rnaseq variant call set. Importantly, allele frequencies in our study were significantly positively correlated with the allele frequency of germline variants listed in the population gnomAD database, supporting the reliability of our calls. Specifically, the allele frequency in GnomAD correlated (Pearson’s correlation coefficient) with wxs-normal variant set (r > 0.963), wxs-tumor variant set (r > 0.964), rnaseq-tumor variant set (r > 0.937) and combined-wxs-rnaseq variant set (r > 0.947) (Chatrath et al., 2019).

Once the genotype status for each variant in each patient is identified, the user can check the survival outcome of minor allele compared to major allele for each variant. The Kaplan-Meier survival analysis for the three genotype (Figure 3) in variant rs1131397 at chromosome 1 and 154965759 locus reveals that patients with homozygous alternate genotype (CC) and heterozygous genotype (GC) have significantly (p-value = 7.48e-04) worse prognosis than the reference genotype (GG), determined using log rank test. In addition, the multivariate Cox regression analysis in Figure 4 reveals that minor allele in rs1131397 is an independent factor for predicting LGG patient overall survival after adjusting for confounding clinical risk factors.

## Limitations

### Computational resources

This protocol performs variant calling, pre-processing VCFs, calculating sequencing coverage, determining variant status, merging variant status from multi-samples, determining genotype of variant with low sequencing coverage from the respective wxs-normal, wxs-tumor and rnaseq-tumor BAM files. As these calculations are computationally intensive, we recommend running the protocol on a high-performance cluster. The memory and number of CPUs required to run each step (See Step-by-step method details) may vary with the number of samples, size of the dataset, sequencing depth, and size of the capture area. Smaller datasets will have lower memory requirements and so this method can be used on them with limited computational resources. This protocol requires the user to modify the memory and CPUs needed in the job script for each step accordingly.

### Data access

A token is required for downloading controlled test dataset (See Before you begin) from the GDC. To obtain access to controlled dataset requires an NIH eRA Commons account and then dbGaP authorization. Instructions on how to apply for GDC controlled dataset access can be found at https://gdc.cancer.gov/access-data/obtaining-access-controlled-data.

### Computational time

Downloading GDC datasets and germline variant calling consumes considerable amount of time and computational resources. We recommend processing a small number of wxs and rnaseq BAM files to guide the computational resources requirement for the future analyses. This protocol requires user to modify the time needed in the job script for each step accordingly.

### Basic programming knowledge

This protocol follows SLURM based schema and requires knowledge of the bash and R scripting language. Newer users may need to learn some basic Linux commands such as ls, echo, cd, rm, mv, mkdir, find, nano, awk, sed, grep, pwd, bash, and sbatch used in this protocol to execute it fully.

## Troubleshooting

### Problem 1

A common warning may be displayed for the bam index: BAM index file is older than BAM file (corresponding protocol step: Part 1: Variant calling using VarDictJava; step 1g).

**Figure:**
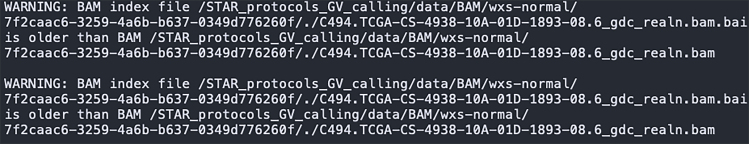

### Potential solution

It is a warning message for bam index file. It can be ignored if you are sure that the index file is up to date. You can create the more recent index file of the bam file using the command below and the warning message should go away.

>samtools index ∗.bam

### Problem 2

A common error may be displayed: These module(s) or extension(s) exist but cannot be loaded as requested (corresponding protocol step: Part 1: Variant calling using VarDictJava; step 1g).

**Figure:**
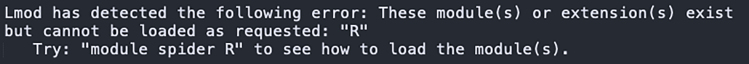

### Potential solution

The module system may vary between Linux clusters. The user needs to know how to load and unload modules in their online Linux cluster. For example, to search how to load R modules on our cluster, use the below command.

>module spider R

***Note:*** This protocol expects that R, VarDictJava, SAMtools and BCFtools are installed on your local or online Linux cluster and loaded in the system PATH.

### Problem 3

Fatal error: cannot open file ‘extract_uniqueVariants.R’: No such file or directory (corresponding protocol step: Part 2: Preprocessing of VCF files; step 4).

### Potential solution

Try to set the full path of the R script in the extract_uniqueVariants.sh bash script.

### Problem 4

slurmstepd: error: ∗∗∗ JOB ID CANCELLED DUE TO TIME LIMIT ∗∗∗ (corresponding protocol step: Part 5: Creating multi-sampled variant status file; step 9c).

### Potential solution

This error will be in the error output file. This error occurs when the job which was running on the Linux cluster may have reached the maximum time limit as requested in the job script and thus canceled. Try increasing the time limit in the job script with the --time= sbatch flag.

### Problem 5

Error in names(x) < value: ‘names’ attribute must be the same length as the vector (corresponding protocol step: Part 6: Insert variant call for unknown variant status in normal samples; step 15c).

### Potential solution

This error message suggests that Sample_Barcode in the patient_list does not match with the sample names in the processed_CombinedPotentialSNPs.txt file. The Sample_Barcode should match exactly with the sample names in the processed_CombinedPotentialSNPs.txt file for each data type.

## Resource availability

### Lead contact

Further information and requests for resources should be directed to and will be fulfilled by the technical and lead contacts, Divya Sahu (dsahu@uab.edu) and Anindya Dutta (duttaa@uab.edu).

### Materials availability

This study did not generate new unique reagents.

## Data Availability

The data/analyses presented in the current protocol have been deposited in and are available from the dbGaP database under dbGaP accession phs000178.v11.p8 https://www.ncbi.nlm.nih.gov/projects/gap/cgi-bin/study.cgi?study_id=phs000178.v11.p8. The code is available on GitHub and can access at https://github.com/ds21uab/STAR_protocols_GV_calling; https://doi.org/10.5281/zenodo.6127363
